# Punctuated growth of InAs quantum dashes-in-a-well for enhanced 2-μm emission

**DOI:** 10.1186/s11671-023-03810-y

**Published:** 2023-03-06

**Authors:** R. J. Chu, Y. Kim, S. W. Woo, W. J. Choi, D. Jung

**Affiliations:** 1 Center for Opto-Electronic Materials and Devices, Korea Institute of Science and Technology, Seoul, 02792 South Korea; 2 Division of Nanoscience and Technology, University of Science and Technology, Seoul, 02792 South Korea; 3 Department of Materials Science and Engineering, Korea University, Seoul, 02841 South Korea; 4 Department of Materials Science and Engineering, Research Institute of Advanced Materials, Seoul National University, Seoul, 08826 South Korea

## Abstract

InAs quantum dashes (Qdash) engineered to emit near 2 μm are envisioned to be promising quantum emitters for next-generation technologies in sensing and communications. In this study, we explore the effect of punctuated growth (PG) on the structure and optical properties of InP-based InAs Qdashes emitting near the 2-μm wavelength. Morphological analysis revealed that PG led to an improvement in in-plane size uniformity and increases in average height and height distribution. A 2 × boost in photoluminescence intensity was observed, which we attribute to improved lateral dimensions and structural stabilization. PG encouraged formation of taller Qdashes while photoluminescence measurements revealed a blue-shift in the peak wavelength. We proposed that the blue-shift originates from the thinner quantum well cap and decreased distance between the Qdash and InAlGaAs barrier. This study on the punctuated growth of large InAs Qdashes is a step toward realizing bright, tunable, and broadband sources for 2-μm communications, spectroscopy, and sensing.

## Introduction

2-μm light emitters and detectors are poised as crucial components for next-generation telecommunication systems [1, 2]. Currently, telecommunication is implemented in the 1.55-μm band due to loss considerations in the silica fiber. However, the looming capacity crunch [3] in data and telecommunication necessitates the development of new materials and devices to increase data transmission rates. One such innovation is the hollow-core photonic bandgap fiber, a low-loss, low-nonlinearity alternative to silica fibers. The discovery that these new fibers have a minimum loss at around 2 μm has opened the possibility for 2-μm-based communication [2, 4, 5], the full realization of which will demand parallel and reinvigorated research on light emitters in the 2-μm spectral band. Beyond communication, these emitters can also complement the development of lidar systems [1], gravitational wave observation [6], and planetary gas sensing [7].

Commercial semiconductor laser diodes emitting near 2 μm usually employ epitaxially-grown quantum wells (QW) grown on GaSb [8]. For data and telecommunication laser diodes, however, InP has been the choice of substrate due to its relatively more mature infrastructure for large-scale manufacturing. There have been a few studies on InP-based lasers emitting at 2 μm and beyond, but these low-bandgap QW lasers suffer from low gain and high-temperature sensitivity, limiting their practical use [9, 10]. At the shorter O-band (1.3 μm), InAs quantum dots (QDs) grown on GaAs are established to be superior quantum emitters compared to their bulk and QW counterparts due to their 3D carrier confinement, Dirac delta-like density of states, and dislocation tolerance [11, 12]. Quantum emitters at 2 μm would benefit greatly from the advantages of zero-dimensionality, but 2 μm is beyond the reach of the conventional InAs QDs on GaAs system.

InP-based InAs nanostructures generally grow into elongated structures under most growth conditions; hence, they have been referred to as quantum dashes. Due to the lower lattice mismatch and bandgap difference compared to the InAs QD/GaAs system, InAs Qdashes can be grown larger with longer emission wavelengths. Thus, the InAs Qdash/InP system has been heavily studied for the technologically important C-band (1.55 μm) [13–15]. Placing the InAs Qdashes between InGaAs QWs (QDaWell) further weakens the quantum confinement effect, which finally enables 2-μm emission [16]. This, however, entails growing large nanostructures near or beyond their elastic limit, which could then relax and generate dislocations, ultimately lowering the quantum efficiency of the device.

Reports on the photoluminescence of InP-based InAs Qdashes emitting in the 1.8–2.1-μm range are relatively sparse [17–22]. In most cases, the QDs are grown using metal organic vapor phase epitaxy, which could lead to blue-shifted emission due to heat-induced In-Ga intermixing during the growth of the top device layers. Furthermore, the emphasis has been on achieving longer emissions rather than the study of growth strategies to increase Qdash luminescence. To realize highly performing lasers, there is still room in optimizing the growth of InAs Qdashes, especially for the larger Sb-free 2-μm emitting InAs QDaWells. One strategy to improve the luminescence from QDs and Qdashes is introducing a growth interruption or ripening stage, which provides time for the adatoms to migrate into crystalline islands [23, 24]. A 60 s growth interruption has been shown to lead to up to 400% boost in photoluminescence intensity [24]. A modification of this strategy was adopted by Mukhametzhanov et al*.*, who subdivided the InAs deposition into stages, with growth interruptions between deposition steps [25]. In their analysis, incoming In adatoms preferentially incorporate into smaller islands during the growth interruption, thus improving size uniformity and photoluminescence intensity. This so-called punctuated island growth was then used to grow GaAs-based InAs QD infrared photodetectors [26]. A similar approach was adopted by Rödel et al*.* in the growth of GaAs-based InP QDs [27]. Their strategy consisted of submonolayer depositions separated by 20 s growth interruptions. This strategy led to an increase in QD height, increase in height dispersion, and decrease in QD density. The morphological changes were also found to affect the optical properties of the QDs. To the best of our knowledge, there are no reports yet on the PG of InP-based InAs QDs or Qdashes.

In this study, we explore the PG of InAs Qdashes on InP for emission near the 2-μm spectral window. We show the effects of punctuated growth on the photoluminescence of Qdashes and correlate them with changes in the structure of and confinement levels in the QDaWell active region. With these results, we believe that the punctuated growth of large InAs quantum dashes is a step toward realizing bright tunable broadband sources for 2-μm communications, spectroscopy, and sensing.

## Experimental section

**Fig. 1:**
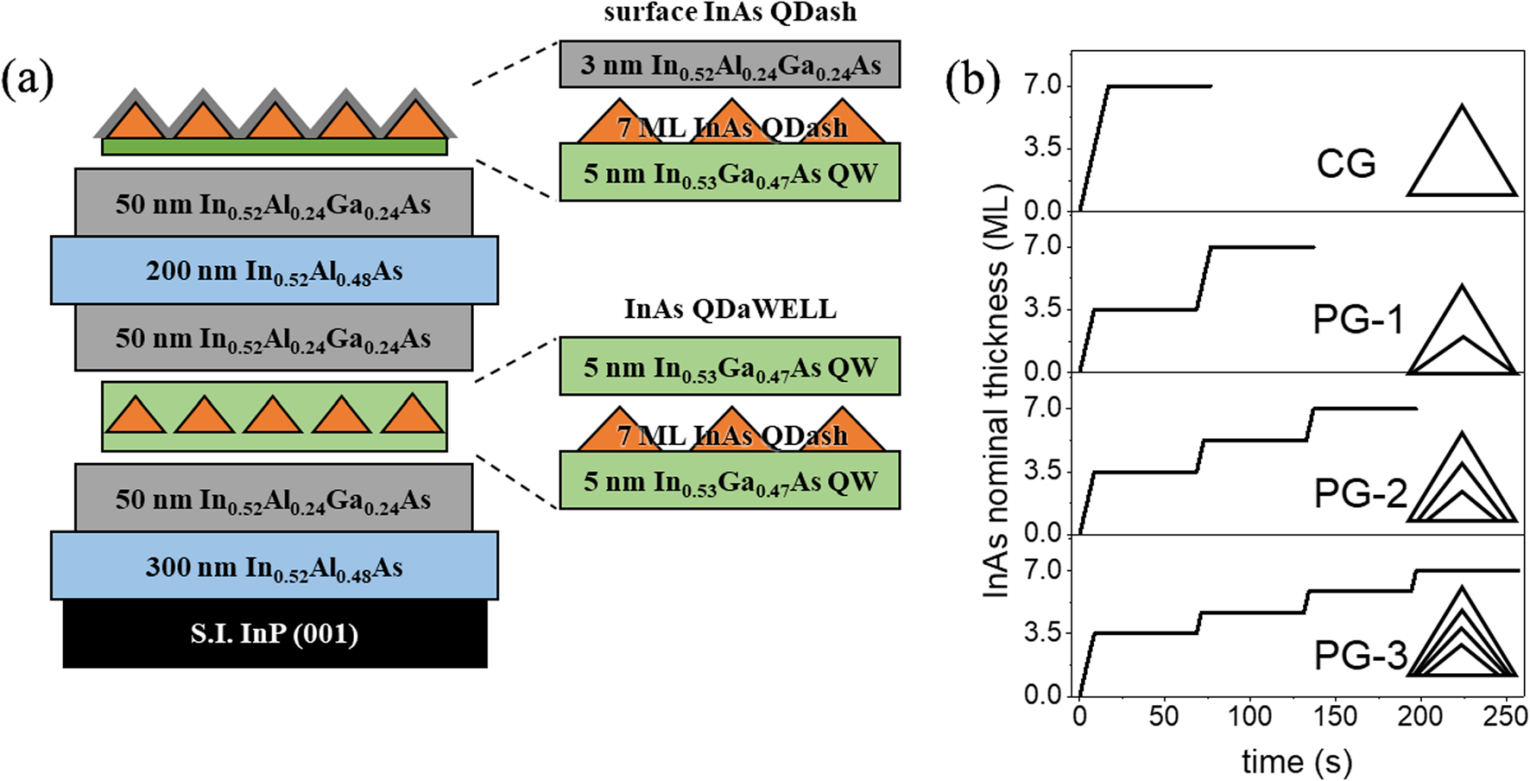
**a** Schematic structure of the InAs quantum dashes. **b** Punctuated growth scheme of the InAs quantum dash active region

The samples were grown on semi-insulating InP wafers in a Veeco GEN930 solid-state molecular beam epitaxy chamber equipped with an in-situ reflection high-energy electron diffraction (RHEED) system. The native oxide was removed thermally, then the structure shown schematically in Fig. 1a was grown. First, a 300 nm In_0.52_Al_0.48_As buffer and a 50 nm In_0.52_Al_0.24_Ga_0.24_As digital alloy layer were grown. Then, a quantum dash-in-a-well (QDaWell) region was grown, consisting of 7 ML InAs sandwiched between 5 nm In_0.53_Ga_0.47_As QWs. Then, the following layers were grown: 50 nm In_0.52_Al_0.24_Ga_0.24_As, 200 nm In_0.52_Al_0.48_As, and 50 nm In_0.52_Al_0.24_Ga_0.24_As. Finally, another Qdash layer was grown for AFM analysis, consisting of a 5 nm In_0.53_Ga_0.47_As QW, 7 ML InAs Qdash, and 3 nm In_0.52_Al_0.24_Ga_0.24_As capping to suppress post-growth transformation during sample cooling [24]. As_2_ mode was used during the entire growth sequence. The active region was grown at 500 °C, while the other layers were grown at 500–510 °C based on pyrometer readings. The growth rates for InAs, In_0.53_Ga_0.47_As, and In_0.52_Al_0.48_As were 0.418 ML/s, 2.206 A/s, and 2.241 A/s, respectively. The V/III flux ratios used for In_0.52_Al_0.48_As, In_0.53_Ga_0.47_As, InAs, punctuation, and final ripening were 25, 25, 20, 20, and 5, respectively. These flux ratios were optimized in previous experiments (not shown).

To study the effect of punctuated growth, four samples were grown with different punctuations strategies. A one-step punctuated growth (PG-1) was done on one sample, with a 60 s growth interruption at the 3.5 ML point, at which point the Qdashes have already formed based on the RHEED patterns. Another sample with 2-step punctuated growth (PG-2) was punctuated at the 3.5 and 5.25 ML points. The PG-3 sample was punctuated at the 3.5, 4.67, and 5.83 ML points. As reference, a sample with continuously-grown (CG) Qdashes was also grown. The growth strategy is shown schematically in Fig. 1b. During the final growth interruption in all samples, the V/III flux ratio was lowered to 5 to enhance adatom mobility [23]. All samples were subjected to the same growth conditions after independent optimization of growth temperature, buffer annealing, quantum well annealing, and V/III flux ratio (not shown).

The surface was characterized using a Park Systems atomic force microscope under contact-mode. Photoluminescence (PL) spectra were obtained using a 532-nm laser modulated by an optical chopper and an extended InGaAs photodetector (Thorlabs PDA10DT) connected to a lock-in amplifier. Low-temperature PL spectra were obtained by loading the samples in a cryostat chamber cooled by a He cryo-compressor.

## Results and discussions

**Fig. 2:**
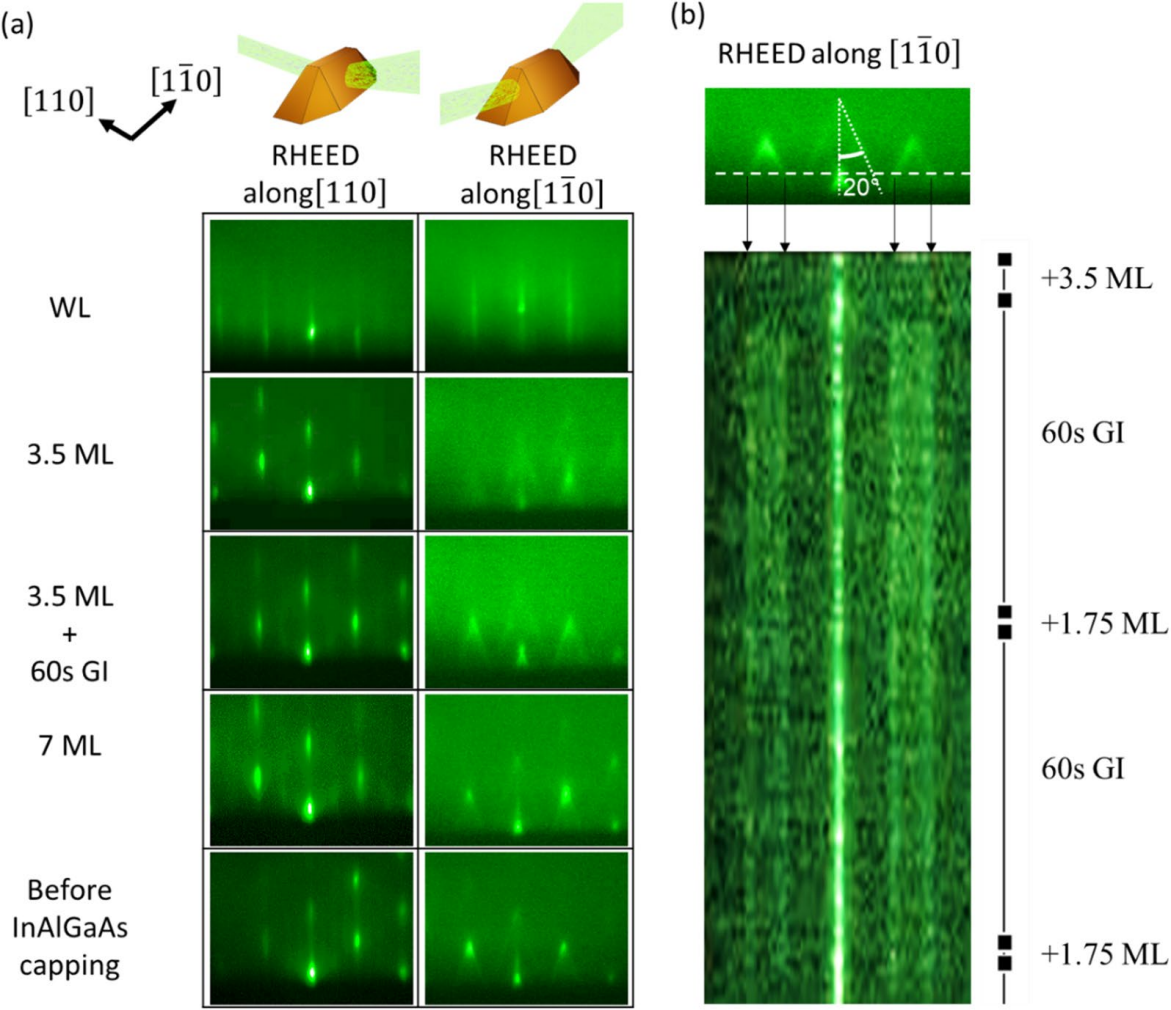
**a** Reflection high-energy electron diffraction (RHEED) images obtained along the $\left[110\right]$ and $\left[1\overline{1 }0\right]$ at different instances of the quantum dash growth. **b** Time series of a single row (marked with the white line) in the RHEED images along $\left[1\overline{1 }0\right]$ during the growth of the PG-2 sample

Figure 2 shows RHEED images obtained in situ during the growth of the buried Qdashes. The RHEED patterns observed along orthogonal directions indicated anisotropy in the morphology of the Qdashes [28]. Along [110], the RHEED patterns show transmission diffraction spots typical of QDs. Along $\left[1\overline{1 }0\right]$, chevron tails emerge, which indicates faceting of the Qdash sidewalls. Previous AFM and TEM studies on InAs QDs and Qdashes have confirmed a remarkable link between the sidewall facets and the angle of the chevron tail with respect to the [001] direction [16, 28, 29]. In our RHEED images, the angle is ~ 20° angle, which corresponds to the {114} family of planes. Figure 2b shows the time evolution of a horizontal line in the RHEED images along $\left[1\overline{1 }0\right]$ obtained during the growth of PG-2 sample. This position was chosen to monitor the angle of the chevron tails. The four vertical lines indicated by arrows in the stacked image means that the chevron tail angle is conserved throughout the growth. This angle is roughly conserved during InAs deposition above 3.5 ML, throughout the entire growth interruption where shape evolution is expected, and regardless of the punctuation strategy (CG to PG-3). Previously, we have also shown that small and large Qdashes have roughly the same aspect ratios [16]. This aspect ratio conservation points to the stability and energetic favorability of the {114} facet [30]. In InAs QDs on GaAs, the stable facet is {136}, which would appear as ~ 29° chevron tails on RHEED images [29].

**Fig. 3:**
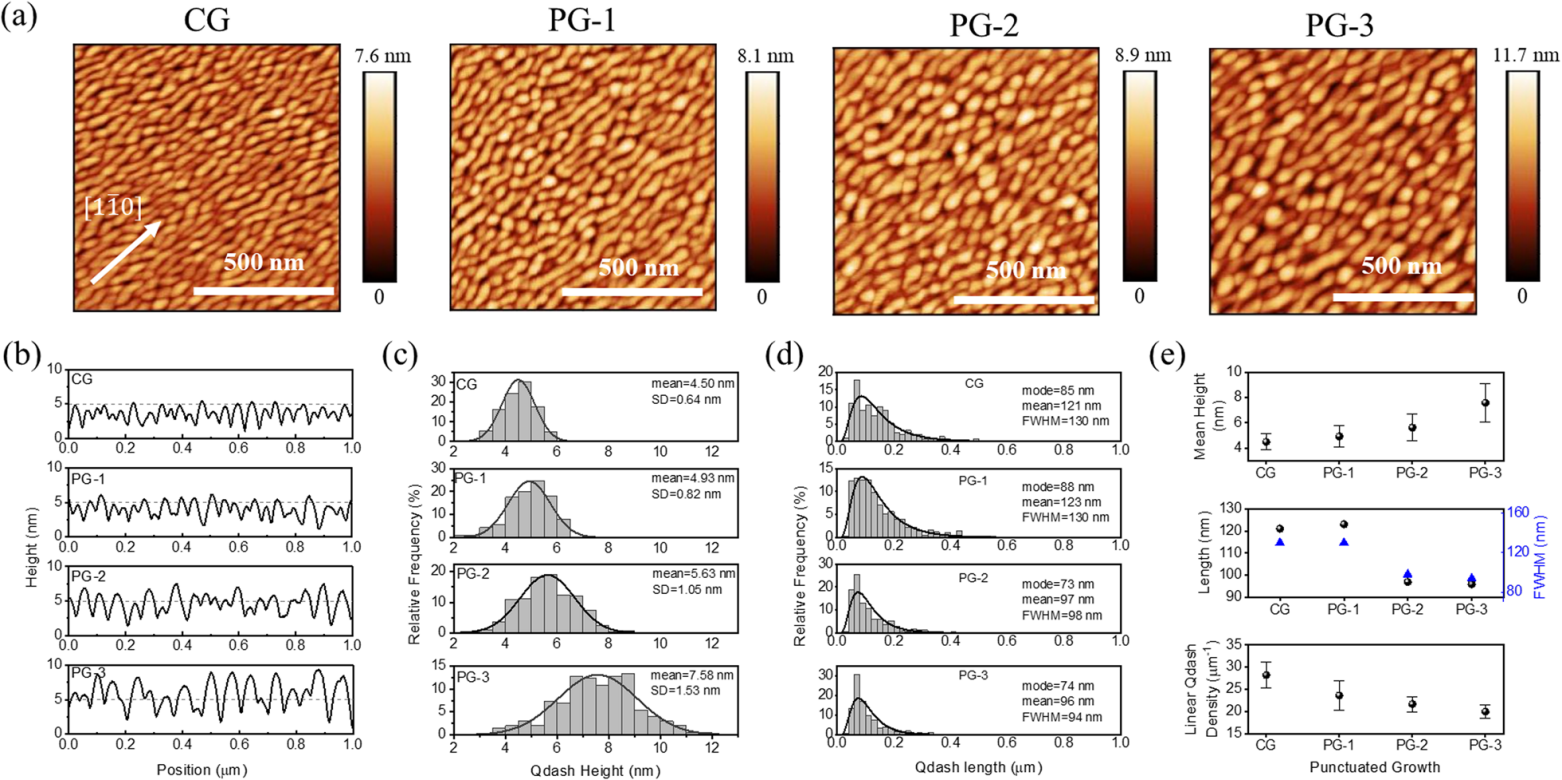
**a** Atomic force microscope (AFM) images of the surface quantum dashes capped by InAlGaAs.** b** Representative line profiles along $\left[1\overline{1 }0\right]$ direction obtained from the AFM scans.** c** Distribution of quantum dash heights.** d** Distribution of quantum dash lengths.** e** Summary of average height (top), length (middle), and linear quantum dash density (bottom)

Figure 3a shows 1 × 1 μm^2^ images of the Qdashes capped by 3 nm InAlGaAs. The elongation seen in the AFM measurements confirms the RHEED observations earlier, revealing Qdashes elongated along the $\left[1\overline{1 }0\right]$ crystal direction as shown in Fig. 3a. The anisotropy is due to slower In adatom diffusion along the $\left[1\overline{1 }0\right]$ direction, which has been attributed to asymmetric surface bonds in the zinc blende structure of InP and In(Al,Ga)As crystals under As-stabilized conditions [31].

Figure 3﻿b features representative line profiles along [110] extracted from the AFM scans. It is already apparent that PG leads to taller and wider Qdashes. The Qdash height distributions are shown in Fig. 3c. A few trends can be noticed. First, the height distribution fits well with a unimodal normal distribution. Note that this will not be the case for buried Qdashes since the upper QW and InAlGaAs barrier will set an upper limit. Second, the mean Qdash height increases from 4.50 nm (CG) to 7.58 nm (PG-3). Lastly, the standard deviation (SD) increases, which indicates a reduction in height uniformity. The parameters are summarized in Fig. 3e (top).

We then look at the distribution of Qdash lengths shown in Fig. 3d. Note that there is some uncertainty in these measurements because the boundary between our Qdashes is sometimes hard to define. Nevertheless, certain trends emerge. First, the distributions are all unimodal which can be fitted with a lognormal distribution [32]. For CG, there are Qdashes as long 0.92 μm but these are few and far between. Meanwhile, the number of long Qdashes is reduced in PG samples. Second, the mean (modal) length decreases from 121 nm (85 nm) for CG to 96 nm (74 nm) for PG-3, but we observe that the values are similar for CG and PG-1, and for PG-2 and PG-3. Third, the full width at half-maximum (FWHM) shows a decrease from 130 to 94 nm. Here, we selected FWHM instead of SD since it is a more intuitive measure of the distribution’s width. These measurements indicate that the Qdashes become shorter with better uniformity in length. The parameters are summarized in Fig. 3e (middle).

The width distribution is harder to describe due to width fluctuations along the elongation direction. Here, we instead measure the linear Qdash density to roughly estimate the distribution along [110] (Fig. 3e, bottom). The linear density decreases from 28 Qdashes/μm (CG) to 20 Qdashes/μm (PG-3), and the SD also decreases generally. These point to an increase in Qdash width and inter-dash separation. The competing effects of the reduced length and increased width somehow averages out and leads to a Qdash density of 1.3–1.4 × 10^10^ cm^−2^ for all samples.

Taken together, PG leads to an increase in average height and height dispersion, a decrease in length and length dispersion, and increase in width with decreased dispersion. The increase in mean height is only 2 nm, but the decrease in mean (modal) length is 25 nm (11 nm). Hence, this is strong indication that PG-2 and PG-3 Qdashes are more 0D-like in morphology. Meanwhile, the reduction in SD and FWHM in the lateral dimension points to an improved in-plane uniformity. These characteristics are important for their effect on carrier localization.

The observed decrease in uniformity deviates from Mukhametzhanov et al*.*’s study on GaAs-based InAs QDs [25]. However, statistical analysis from AFM images is overall consistent with Rodel et al*.*’s study on cyclic deposition of InP QDs on GaAs [27] and Jung et al.’s study on the growth interruption of InP-based InAs Qdashes [24].

The shape and size evolution of the Qdashes are driven by kinetics and thermodynamics. During the growth interruption, the In adatoms are given time to diffuse and reorder. Along $\left[1\overline{1 }0\right]$, the diffusion is fast, which initially leads to long Qdashes elongated along $\left[1\overline{1 }0\right]$, as is the case for CG. When growth interruptions are introduced, enough time is provided for the In atoms to migrate along the slower $\left[1\overline{1 }0\right]$ direction, leading to the splitting of long Qdashes. Assuming an Ostwald ripening-like mechanism, In adatoms incorporate into larger Qdashes and small Qdashes are consumed by this process. This manifests as an increase in height and width. Meanwhile, the stability of the {114} facets leads to a preservation of aspect ratio along the $\left[1\overline{1 }0\right]$ direction.

**Fig. 4:**
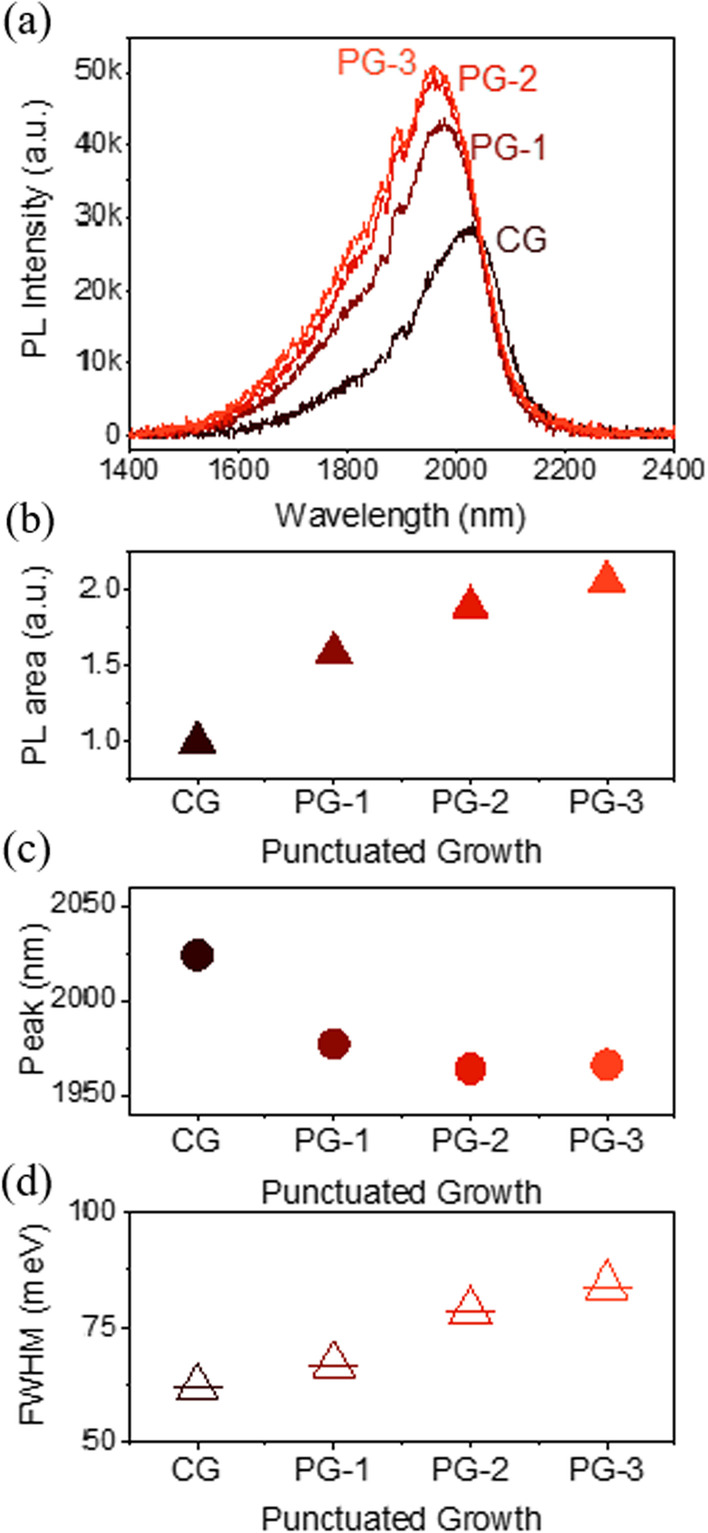
**a** Room temperature photoluminescence spectra of the quantum dashes grown with different punctuation strategies and their corresponding **b** *λ*-integrated intensity, **c** peak wavelength and **d** full-width at half maximum values

Room-temperature photoluminescence spectra are shown in Fig. 4a. Punctuated growth led to up to a twofold enhancement in the *λ*-integrated PL intensity (Fig. 4b). Growth interruptions provide enough time for the Qdashes to approach their equilibrium state, with an overall minimization in strain and surface energies, and this contributes to the enhancement in PL intensity [33, 34]. AFM analyses show that PG induces structural transformation, which is assumed to be toward an equilibrium structure. In addition to this, we believe that that the enhancement is also partly due to the reduced lateral dimensions of PG samples as described earlier. Shorter Qdashes approach the 0D case where carriers are localized within a single Qdash and increase the probability of radiation recombination. The ground state emission from CG sample is at 2.02 μm (Fig. 4c). Introducing PG immediately leads to a blue-shift of ~ 18 meV, with the PG-1 at 1.98 μm and PG-2 at 1.96 μm, at which point the blue-shift seems to have saturated. The FWHM values (Fig. 4d) are large, characteristic of the inherent size nonuniformity of Qdashes. Furthermore, there is an increase in the FWHM with more PG, consistent with the increasing height dispersion described previously (Fig. 3c). Despite the increase in FWHM, there is still an increase in intensity. The increase in FWHM is not always a disadvantage. Studies comparing Qdash and QW lasers have shown that Qdashes can have a broader linewidth, but superior lasing performance owing to their quasi-zero dimensionality [17, 35]. Furthermore, the effect of inhomogeneous broadening on Qdashes is expected to be less severe as on QDs [36]. Comparing the results in Ref. [36] and our calculated SD values, we estimate that compared to CG, PG-1 will have a ~ 4% decrease in peak gain coefficient, while PG-3 will have ~ 10% reduction. This reduction could be compensated by the improved in-plane dimensions and potentially improved thermal lasing properties. Hence, PG Qdashes are expected to have better 0D-related lasing properties than the CG Qdashes owing to their morphology.

**Fig. 5:**
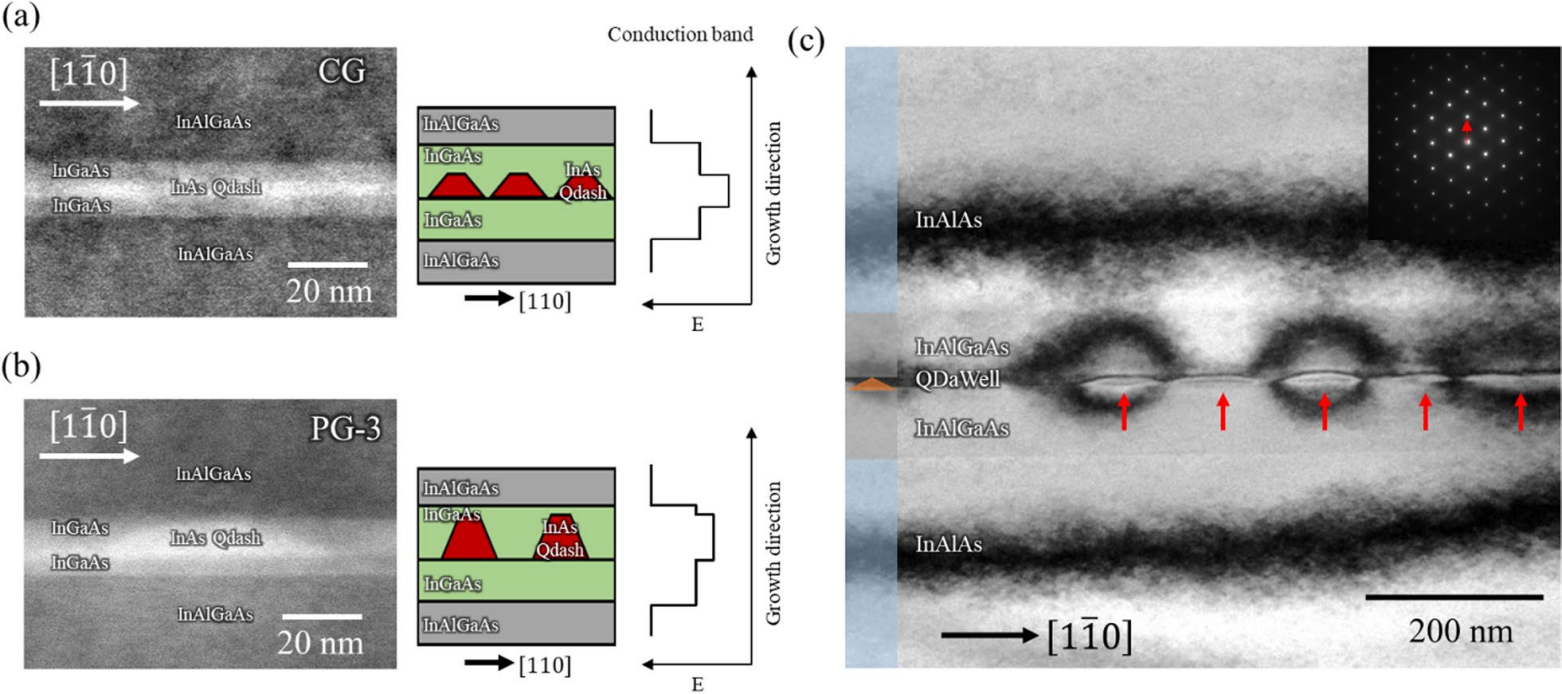
**a**, **b** Scanning transmission electron microscope images, schematic illustration and band alignment of the **a** CG and **b** PG-3 quantum dashes. **c** Bright-field TEM image of the PG-3 sample under (002) two-beam condition and corresponding selected area electron diffraction image. Qdashes are labeled with the red arrows

The blue-shift appears to be contrary to the increase in Qdash height observed by the AFM measurement above. Taller QDs and Qdashes are expected to exhibit a red-shift due to the reduced quantum confinement effect. Two common causes of blue-shift are In desorption during the growth interruption and Ga intermixing during prolonged high-temperature growth, but both of these are unlikely to be the cause. In desorption is unlikely due to the presence of high arsenic species and the modest temperature used. Furthermore, AFM clearly shows bigger Qdashes in PG samples despite the longer growth interruptions which would’ve led to a redshift. Ga intermixing with the QWs is also a possibility, but we do not expect this amount of blue-shift with the temperature and duration in our growth. Instead, we propose that the blue-shift is primarily due to the relative thinning of the QW cap due to the increasing Qdash height and increasing proximity of the InAlGaAs barrier. Figure 5a, b shows scanning transmission electron microscope (STEM) images obtained with $\left[1\overline{1 }0\right]$ in the lateral direction. In the CG samples, the Qdashes appear fully buried by the 5 nm post-QW. But in the case of the PG-3 samples, the tip of the Qdashes have become very close and even in contact with the InAlGaAs barrier. Hence, taller Qdashes will experience a stronger quantum confinement which leads to a larger ground state energy. The schematic structure and band alignment of CG and PG QDaWells are schematically shown in Fig. 5a, b. In a separate experiment (see SI), we have found that 6ML InAs QDaWell (QW + Qdash + QW) sample had an 18 meV lower energy than a sample without the post-QW (QW + Qdash). This further provides credence to our hypothesis.

The increasing Qdash height raises the question of strain relaxation and dislocation formation. In our prior experiments, PL intensity starts to decrease at an InAs nominal thickness of 8 ML [16], which we interpreted as the onset of dislocation formation. In the present study, the Qdashes are much taller than the 8ML Qdashes, and dislocations might have started to form. We performed bright-field TEM observations under (002) two-beam condition to increase dislocation contrast. Figure 5c show representative bright-field TEM image from the PG-3 sample. No dislocations were observed from any of the samples, suggesting that the tall Qdashes remained coherent after punctuated growth.

**Fig. 6:**
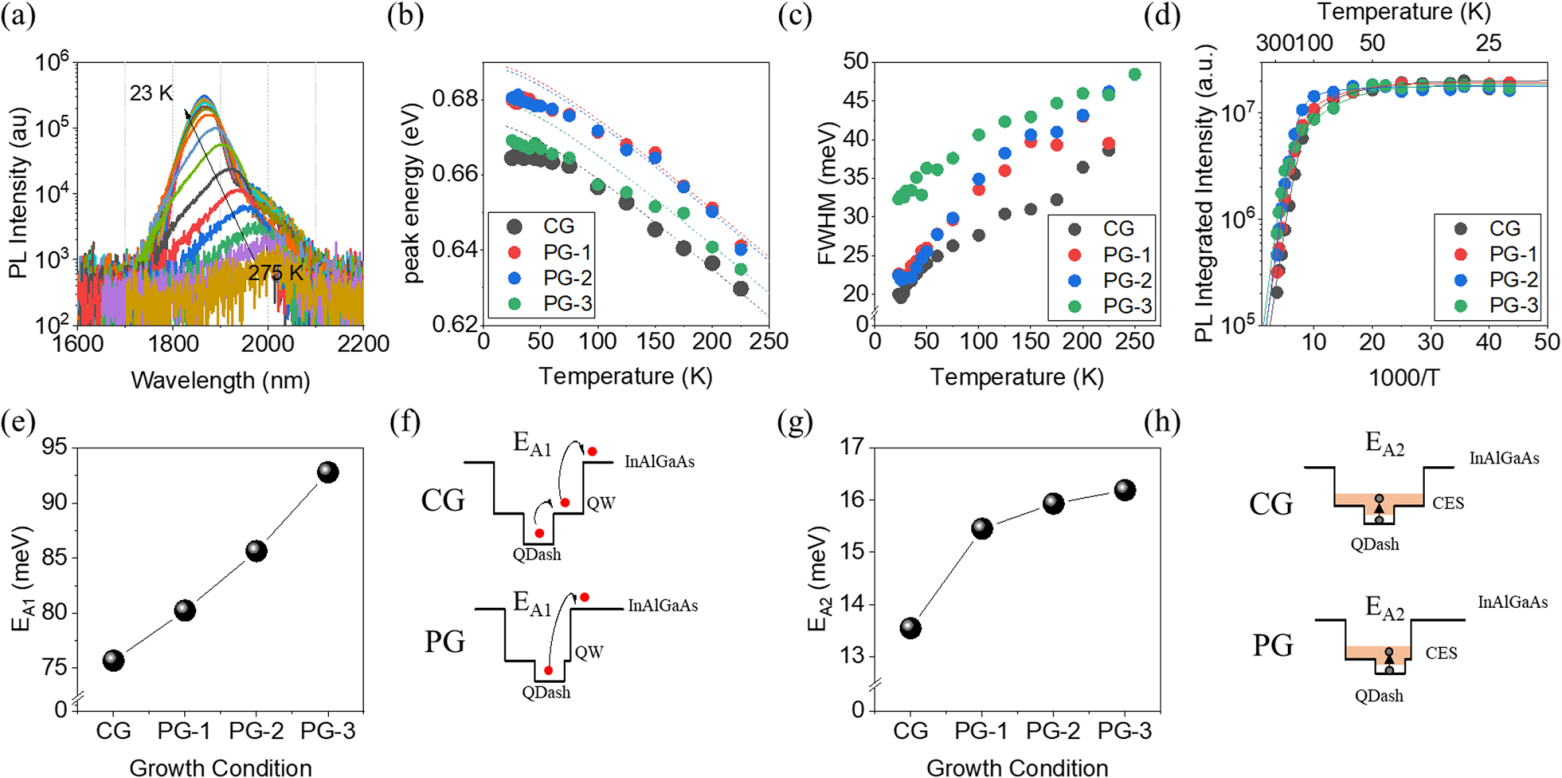
**a** Low temperature photoluminescence spectra for the PG-3 quantum dashes. **b**–**d** Temperature-dependence of the **b** peak wavelength, **c** full-width at half-maximum value, and **d** integrated intensity plotted against 1000/T with the fitted curve. **e**–**h** Activation energy trends and schematic diagrams illustrating the origin of the trends

We also performed low-temperature photoluminescence to gain insight into the carrier dynamics. Figure 6a shows the temperature-dependent PL spectra from the PG-3 sample. For all samples, there is a blue-shift (Fig. 6b) at decreasing temperatures. The high temperature data (> 150 K) were fitted with the Varshni equation using parameters from bulk InAs. Whereas QDs show an S-curve trend due to carrier redistribution and thermoactivation, Qdashes behave more like QWs due to their reduced carrier localization [17]. It is only below 75 K where the peak intensities deviate noticeably from the Varshni model. A closer look at the PG samples reveals a weak curve at around 75–125 K, which is an indication that PG Qdashes have reduced dimensionality. The FWHM values (Fig. 6c) increase with temperature, consistent with previous studies on Qdashes [17]. The increased FWHM of PG-3 is a consequence of the height dispersion discussed earlier. In InAs QDs on GaAs, the FWHM exhibits a V-shape trend [37]. However, for the QDaWell structure, the FWHM increases with temperature because their lower spatial confinement makes thermally-assisted carrier distribution possible even at low temperatures.

The integrated PL intensity (Fig. 6d) quenches at higher temperature due to the thermal escape of carriers [38]. From the temperature-dependence of the PL intensity, we obtained thermo-activation energies and plotted them in Fig. 6e. The Arrhenius curve was fitted using Eq. 1.

1 $$I\left( T \right) = \frac{1}{{1 + A_{1} \exp \left( { - \frac{{E_{{{\text{A}}1}} }}{kT}} \right) + A_{2} \exp \left( { - \frac{{E_{{{\text{A}}2}} }}{kT}} \right)}}$$

The larger activation energy *E*_A1_ is associated with barrier height, while the smaller energy *E*_A2_ is associated with inter-dot transfer via coupled excited states or wetting layer states. As more PG is introduced, *E*_A1_ increases (Fig. 6e), suggesting higher barrier energy. This is due to the thinning of the post-QW and increasing proximity of the InAlGaAs barrier as postulated earlier. As more PG is introduced, *E*_A2_ also increases (Fig. 6g). In previous studies on smaller Qdashes, the *E*_A2_ has been attributed to the excitation of carriers, usually the hole, to an excited state or wetting layer state and subsequent transfer to neighboring Qdashes via these coupled states [38–40]. As these values are much lower than the separation between the ground and excited states, this activation channel may be assumed to be a transition between hole states prior to carrier distribution via coupled high energy states. The larger energy barrier in PG3 due to the thin upper QW could increase the hole energy spacings and lead to the observed trend. However, the small difference in the values may as well be within bounds of uncertainty. If that were the case, then this activation channel may be considered invariant with PG strategy. Others have noted that identifying the mechanism behind this *E*_A2_ energy is not a straightforward process [40]. Determining the exact energy levels may elucidate this, but measurement of PL in this 2 μm is inherently noisy. This may also be elucidated by theoretical modeling, but it is beyond the scope of our work.

## Conclusion

Punctuated growth is a useful tool in the epitaxial growth of InAs Qdashes on InP for improving emission intensity at the 2-μm spectral wavelength and beyond. In combination with optimizations in growth temperature, multi-step annealing, and group V/III ratio, punctuated growth was found to lead to a twofold increase in PL intensity, which is promising for high performance LEDs and lasers. We observed an increase in emission linewidth and a blue-shift in PL emission wavelength, which we attributed to the changes in morphology and quantum confinement effect. The origin of the blue-shift was proposed to be due to the thinning of the QW cap and increasing proximity of the InAlGaAs barrier. These results show that punctuated growth is a promising technique for realizing high-quality quantum emitters in the 1.8–2.1-μm range for telecommunication and sensing applications.

## Data Availability

The datasets used and/or analyzed during the current study are available without restriction from the corresponding author on reasonable request.

## Abbreviations

QD: Quantum dot
QW: Quantum well
Qdash: Quantum dash
QDaWell: Quantum dash-in-a-well
PG: Punctuated growth
RHEED: Reflection high energy electron diffraction
AFM: Atomic force microscopy
SD: Standard deviation
PL: Photoluminescence
SEM: Scanning electron microscope
TEM: Transmission electron microscope
FWHM: Full-width at half maximum
